# The Autoimmune Gastritis Puzzle: Emerging Cellular Crosstalk and Molecular Pathways Driving Parietal Cell Loss and ECL Cell Hyperplasia

**DOI:** 10.3390/cells14201576

**Published:** 2025-10-10

**Authors:** Sara Massironi, Elena Oriani, Giuseppe Dell’Anna, Silvio Danese, Federica Facciotti

**Affiliations:** 1 Department of Medicine and Surgery, Vita-Salute San Raffaele University, 20132 Milan, Italy; 2 Department of Biotechnology and Biosciences, University of Milano-Bicocca, 20126 Milan, Italy; 3 Gastroenterology and Gastrointestinal Endoscopy Division, IRCCS San Raffaele Hospital, Via Olgettina 60, 20132 Milan, Italy; dellanna.giuseppe@hsr.it; 4 Gastroenterology and Gastrointestinal Endoscopy Division, IRCCS Policlinico San Donato, Piazza Edmondo Malan 2, 20097 San Donato Milanese, Italy

**Keywords:** autoimmune gastritis, parietal cells, hypergastrinemia, ECL cell hyperplasia, gastric neuroendocrine neoplasms, ER stress, autophagy, microbiome

## Abstract

Autoimmune gastritis (AIG) is a chronic, organ-specific autoimmune disease characterized by progressive destruction of gastric parietal cells driven by autoreactive CD4^+^ T-cells, epithelial stress pathways, and microbial factors. Parietal cell loss results in achlorhydria, intrinsic factor deficiency, and vitamin B12 malabsorption, ultimately leading to pernicious anemia. Compensatory hypergastrinemia promotes enterochromaffin-like (ECL) cell hyperplasia and contributes to the development of type 1 gastric neuroendocrine neoplasms (gNENs). These clinical consequences are well recognized, yet the cellular and molecular mechanisms driving mucosal atrophy and neoplastic transformation remain incompletely defined. Recent advances highlight the role of endoplasmic reticulum stress, impaired autophagy, innate immune effectors, and dysbiosis in perpetuating inflammation and epithelial injury. The frequent coexistence of AIG with other autoimmune disorders further adds to its clinical complexity. Therapeutic options remain limited, spanning vitamin B12 replacement and endoscopic management to emerging targeted approaches. Netazepide, a gastrin/CCK2 receptor antagonist, is the only agent tested in clinical trials, whereas interventions targeting ER stress, autophagy, immune tolerance, or microbiome composition are still in the preclinical stage. Clarifying these mechanisms is crucial to improve biomarker development, optimize surveillance, and identify targeted therapies to prevent neoplastic transformation.

## 1. Introduction

Autoimmune gastritis (AIG) is a chronic, organ-specific autoimmune disease characterized by progressive destruction of gastric parietal cells. This process leads to hypochlorhydria, intrinsic factor deficiency, vitamin B12 malabsorption, and an increased risk of gastric neoplasia [1,2,3,4,5,6]. While these clinical sequelae are well recognized, the underlying molecular and cellular mechanisms remain only partly understood [2,6,7,8]. The disorder is often underdiagnosed, with a prevalence estimated between 0.5% and 2% in the general population, rising to 5–10% among patients with other autoimmune conditions [1,2,3,4,5,6,7,8].

Central to AIG pathogenesis is the autoreactive CD4^+^ T-cell response against the gastric H^+^/K^+^ ATPase, the proton pump essential for acid secretion [6,7,9,10]. These T-cells drive parietal cell apoptosis via Fas/FasL and perforin–granzyme pathways, amplifying apoptosis and leading to oxyntic atrophy [1,10]. Innate immune cells further amplify tissue damage: NK cells can directly mediate epithelial injury [11], while dendritic cells (DCs) and innate lymphoid cells (ILCs), though less defined in AIG, are likely to influence local immune polarization [11,12,13]. B cells and autoantibodies directed against parietal cells and intrinsic factor serve as useful diagnostic markers, but their pathogenic contribution remains debated [1].

Moreover, epithelial stress responses such as endoplasmic reticulum (ER) stress and defective autophagy may heighten susceptibility to immune-mediated injury, while Fas/FasL signaling provides a mechanistic bridge between immune aggression and epithelial apoptosis [1]. However, direct evidence for ER stress in human AIG remains limited, highlighting an important knowledge gap.

The progressive loss of parietal cells disrupts acid-mediated feedback, leading to compensatory hypergastrinemia. Gastrin acts on CCK2R to stimulate enterochromaffin-like (ECL) cell proliferation, predisposing to type 1 gastric neuroendocrine neoplasms (gNENs) [14,15,16,17,18,19,20]. The downstream signaling nodes, such as ERK/MAPK, PI3K/Akt, and STAT3, sustain proliferation and survival signals, likely cooperating with the inflammatory microenvironment in neoplastic progression [17,21,22,23].In parallel, hypochlorhydria reshapes the gastric niche and is associated with dysbiosis; microbial products engage epithelial and immune receptors (e.g., Toll-like receptors, TLRs), sustaining inflammation and epithelial stress and potentially modulating gastrin biology [24].

Finally, AIG seldom occurs in isolation but frequently coexists with other autoimmune disorders, including autoimmune thyroid disease, type 1 diabetes, and vitiligo, supporting the concept of a shared autoimmune predisposition. This broader context underscores the need to consider AIG not only as an isolated gastric condition but also as part of a systemic autoimmune spectrum [4,7].

This review integrates immune, epithelial, and microbial mechanisms to explain the transition from parietal cell loss to ECL hyperplasia, highlights unresolved questions, and discusses therapeutic opportunities and future research directions.

## 2. Methods

We searched PubMed and Scopus databases for articles published between January 1990 and March 2025. Search terms included “autoimmune gastritis,” “parietal cell,” “hypergastrinemia,” “enterochromaffin-like cells,” “neuroendocrine tumors,” “ER stress,” “autophagy,” and “microbiome.” We included original research articles, translational studies, and reviews in English that addressed immune, epithelial, microbial, and signaling aspects of AIG pathogenesis or clinical outcomes. Case reports, conference abstracts without full data, and studies not directly related to AIG were excluded. Reference lists of the selected articles were also screened manually to identify additional relevant studies.

## 3. Mechanisms of Parietal Cell Loss

Parietal cell loss in AIG reflects the convergence of autoreactive T-cell cytotoxicity, innate immune amplification, and epithelial stress responses. While adaptive immunity represents the initiating force, innate cytotoxicity and impaired epithelial resilience broaden the pathogenic spectrum and highlight potential therapeutic targets (Table 1).

### 3.1. Adaptive Immunity

The autoimmune destruction of parietal cells in AIG is primarily driven by autoreactive CD4^+^ T lymphocytes that recognize epitopes of the gastric H^+^/K^+^ ATPase, the major parietal cell autoantigen. These CD4^+^ T-cells mainly differentiate into Th1 effector producing interferon-gamma (IFN-γ) and tumor necrosis factor-alpha (TNF-α), which amplify inflammation and recruit cytotoxic CD8^+^ T-cells, macrophages, and NK cells [25,26]. IFN-γ also directly induces the upregulation of MHC class II molecules on gastric epithelial cells, increasing their susceptibility to T cell-mediated cytotoxicity [27].

In parallel, autoreactive B cell activation results in the production of autoantibodies directed against both the H^+^/K^+^ ATPase and intrinsic factor. While their role is largely diagnostic, these autoantibodies can trigger the classical complement cascade [26,28,29]. The deposition of complement components, notably C3b, on parietal cells facilitates their opsonization and subsequent clearance by phagocytic cells. Moreover, complement fragments further interact with NK cells, enhancing antibody-dependent cellular cytotoxicity (ADCC), thereby accelerating epithelial attrition [30,31].

Failure of regulatory T-cells (Tregs) to adequately suppress autoreactive CD4^+^ populations likely perpetuates the chronicity of this autoimmune response, though mechanistic data in AIG remain limited [32].

### 3.2. Intrinsic Epithelial Stress Pathways

Parietal cells are not merely passive targets, but exhibit intrinsic vulnerabilities that modulate their survival in an inflammatory microenvironment. Endoplasmic reticulum (ER) stress is a key driver of apoptosis. It arises from the excessive accumulation of misfolded or unfolded proteins within the ER lumen, a process triggered by inflammatory cytokines such as IL-1β and TNF-α. Activation of the unfolded protein response (UPR) seeks to restore ER homeostasis through translational attenuation, upregulation of chaperones like GRP78, and enhancement of ER-associated degradation pathways [33]. However, chronic or overwhelming ER stress shifts the UPR towards pro-apoptotic signaling, primarily mediated by the PERK-eIF2α-ATF4-CHOP axis. CHOP (C/EBP homologous protein) acts as a critical transcription factor that downregulates Bcl-2, promotes Bax activation, and instigates mitochondrial outer membrane permeabilization, culminating in cytochrome c release and caspase cascade activation [34,35].

Simultaneously, death receptor-mediated apoptosis plays a pivotal role in parietal cell loss. The Fas receptor (CD95) is markedly upregulated on parietal cells under inflammatory conditions. Engagement of Fas by its ligand FasL, expressed on infiltrating T-cells and NK cells, triggers the assembly of the death-inducing signaling complex (DISC). This complex recruits and activates caspase-8, initiating the extrinsic apoptotic pathway that converges on effector caspases such as caspase-3, leading to cellular demolition [36].

Autophagy, normally essential for clearing damaged cells [37], is impaired in the inflamed gastric mucosa of AIG. Under physiological conditions, autophagy mitigates cellular stress by removing damaged organelles and aggregated proteins. In AIG, persistent inflammation and oxidative stress compromise autophagic flux, diminishing the capacity of parietal cells to adapt to metabolic and proteotoxic insults [38]. This autophagic insufficiency heightens susceptibility to apoptosis, thereby reinforcing epithelial attrition [38,39].

Oxidative stress represents an additional pathogenic layer, wherein excessive production of reactive oxygen species (ROS) during chronic inflammation inflicts damage on mitochondrial DNA, lipids, and proteins. Mitochondrial dysfunction ensues, characterized by loss of membrane potential and further ROS generation, creating a self-perpetuating loop of oxidative injury and apoptotic signaling [40,41].

### 3.3. Innate Immunity

The innate immune pathways further amplify parietal cell destruction in AIG. Natural killer (NK) cells represent more than 20% of gastric mucosal lymphocytes and can directly mediate epithelial injury. They act via perforin–granzyme release and Fas–FasL interactions [42,43,44], but are particularly effective in the context of antibody-dependent cellular cytotoxicity (ADCC). Parietal cell-specific autoantibodies bound to the H^+^/K^+^-ATPase provide a target for Fc receptor engagement, enabling NK cells to amplify damage initiated by autoreactive T and B cells.

Activation of the classical complement pathway by parietal cell-directed autoantibodies may further exacerbate mucosal injury. Complement deposition promotes opsonization, neutrophil recruitment, and membrane attack complex (MAC) formation, directly lysing parietal cells. Although data in human AIG are limited, evidence of complement activity in gastric tissue supports its contribution as an underrecognized effector mechanism.

Dendritic cells (DCs) in the gastric lamina propria and submucosa may capture and process gastric autoantigens, notably peptides derived from the H^+^/K^+^ ATPase, for presentation via MHC class II molecules to naïve CD4^+^ T-cells in draining lymph nodes. DCs exposed to pathogen-associated molecular patterns (PAMPs) or damage-associated molecular patterns (DAMPs) upregulate co-stimulatory molecules such as CD80 and CD86 and secrete cytokines, including IL-12 and IL-23. IL-12 drives Th1 polarization, while IL-23 fosters Th17 differentiation, contributing to a dual axis of IFN-γ and IL-17-mediated inflammation [45].

Innate lymphoid cells (ILCs), particularly group 3 ILCs (ILC3s), have emerged as pivotal players in mucosal immunity within AIG. ILC3s secrete IL-17 and IL-22, cytokines that modulate the epithelial barrier and immune responses. While IL-22 generally promotes epithelial regeneration, in the context of AIG, the combined action of IL-17 and IL-22 sustains a pro-inflammatory environment that compromises epithelial integrity and perpetuates parietal cell stress. The chronic activation of ILC3s may thus indirectly facilitate the continuous cycle of injury and impaired repair within the gastric mucosa [45].

Together, adaptive autoimmunity, epithelial stress, and innate immunity create a deleterious microenvironment that progressively destroys the parietal cell population [46,47].

However, several aspects remain unresolved. The contribution of dendritic cells and innate lymphoid cells to AIG pathogenesis is largely speculative, as direct experimental evidence is scarce. Likewise, the interplay between complement deposition and NK-mediated cytotoxicity remains underexplored in gastric autoimmunity.

## 4. Hypergastrinemia and ECL Cell Hyperplasia

### 4.1. Gastrin as a Trophic Factor

The depletion of parietal cells results in a profound reduction in gastric acid secretion, which abolishes the negative feedback mechanism regulating gastrin release from antral G cells. As a consequence, gastrin secretion becomes chronically elevated, leading to a state of hypergastrinemia that exerts sustained trophic stimulation on ECL cells located in the oxyntic mucosa [17,20,48,49]. Gastrin exerts its biological effects primarily through binding to the cholecystokinin-2 receptor (CCK2R), a G protein-coupled receptor abundantly expressed on ECL cells [50,51]. Engagement of CCK2R by gastrin triggers intracellular signaling cascades that culminate in ECL cell proliferation, enhanced histidine decarboxylase (HDC) expression, and increased histamine synthesis [21,51]. Histamine, in turn, acts in a paracrine fashion on H2 receptors of parietal cells to stimulate acid secretion; however, in the context of AIG, where parietal cells are depleted, this regulatory axis becomes dysfunctional [52,53].

Chronic hypergastrinemia maintains a persistent proliferative drive on ECL cells, predisposing to the development of linear hyperplasia, nodular hyperplasia, and ultimately dysplastic changes that can progress to type 1 gNENs. This trophic effect is not only quantitative, increasing the number of ECL cells, but also qualitative, potentially altering the differentiation state and secretory profile of these cells [54].

Recent data, however, highlight that gastrin–CCK2R signaling is not exclusively tumor-promoting. In murine models, hypergastrinemia expanded CCK2R^+^ corpus progenitors, accelerated ulcer healing, and mitigated gastric dysplasia during chronic injury [55]. These observations suggest that the biological effects of gastrin are context-dependent, with trophic stimulation contributing both to neoplastic risk and also to mucosal regeneration and repair.

### 4.2. Molecular Pathways in ECL Cell Proliferation

The signaling cascades activated downstream of gastrin–CCK2R interaction are multifaceted and converge to promote proliferation, survival, and eventual neoplastic transformation of ECL cells. One of the principal pathways activated is the extracellular signal-regulated kinase/mitogen-activated protein kinase (ERK/MAPK) pathway. Upon CCK2R activation, adaptor proteins such as Shc and Grb2 recruit Ras, which activates the Raf–MEK–ERK kinase module. Phosphorylated ERK translocates to the nucleus and induces transcription factors, including Elk-1 and AP-1, thereby driving expression of cell-cycle regulators such as cyclin D1 and c-Myc.

In parallel, CCK2R stimulation engages the PI3K/Akt pathway. PI3K generates phosphatidylinositol (3,4,5)-trisphosphate, which recruits and activates Akt. Activated Akt phosphorylates a multitude of downstream substrates that collectively promote cell survival by inhibiting pro-apoptotic factors like Bad and enhancing growth via mTOR signaling. This confers resistance to cell death and sustains the expansion of hyperplastic ECL populations.

Additionally, the crosstalk between PI3K/Akt and JAK/STAT cascades can lead to STAT3 activation, nuclear translocation, and induction of genes promoting proliferation (cyclin D1), survival (Bcl-xL), and angiogenesis (VEGF). Although less directly linked to gastrin signaling, STAT3 appears central to the shift from hyperplasia to neoplasia [56].

Emerging evidence suggests that genetic and epigenetic alterations may further modulate these signaling pathways, lowering the threshold for ECL cell neoplastic transformation. Epigenetic modifications, including DNA methylation and histone acetylation changes, might influence the expression of tumor suppressor genes and oncogenes in ECL cells [57]. Additionally, somatic mutations in components of the signaling pathways or their regulators could confer a growth advantage to ECL cells under the chronic stimulus of hypergastrinemia.

The interplay between hypergastrinemia-induced signaling and the microenvironmental factors, including hypoxia and inflammatory mediators present in the atrophic gastric mucosa, may further synergize to promote the clonal expansion of transformed ECL cells. This multifactorial process underscores the complexity of the progression from hyperplasia to neuroendocrine neoplasia in AIG.

Dysregulated signaling has direct clinical consequences. On one hand, ER stress and defective autophagy accelerate parietal cell apoptosis, exacerbating hypochlorhydria, intrinsic factor deficiency, and pernicious anemia. On the other hand, sustained ERK/MAPK, PI3K/Akt, and STAT3 activation drives ECL proliferation, survival, and resistance to apoptosis, predisposing to type 1 gNENs. Thus, disruption of stress–response and trophic signaling pathways provides the mechanistic link between autoimmune epithelial loss and the two major outcomes of AIG: anemia and neoplasia.

Despite compelling mechanistic hypotheses, clinical validation of ER stress and defective autophagy as drivers of parietal cell loss is still lacking. It remains unclear whether these processes represent primary triggers or secondary amplifiers of autoimmune injury.

## 5. Interplay with the Gastric Microbiome

The gastric microbiome, once thought negligible, is now recognized as a dynamic community shaped by gastric acidity, mucosal immunity, and environmental factors. In the context of AIG, hypochlorhydria fundamentally alters the gastric niche, creating conditions favorable for microbial overgrowth and shifts in microbial composition, collectively termed dysbiosis [24]. The reduction in gastric acidity permits the survival and colonization of bacteria typically restricted to the oropharyngeal cavity and small intestine. This altered microbial milieu is characterized by an increased abundance of non-Helicobacter species, including Streptococcus, Lactobacillus, and Veillonella, which may possess distinct immunomodulatory properties [58]. Moreover, the decreased barrier function associated with epithelial atrophy facilitates closer interactions between luminal microbes and the gastric mucosa, potentially enhancing antigenic stimulation and perpetuating local immune activation [59,60].

One of the critical consequences of microbial dysbiosis in AIG is the potential modulation of innate and adaptive immune responses. Certain bacterial metabolites, such as short-chain fatty acids (SCFAs), can influence the differentiation and function of regulatory T-cells (Tregs) and Th17 cells, thereby skewing the immune balance within the gastric microenvironment. Additionally, microbial-derived pathogen-associated molecular patterns (PAMPs) engage pattern recognition receptors (PRRs) such as Toll-like receptors (TLRs) on epithelial and immune cells, triggering downstream signaling pathways that sustain inflammation and epithelial stress [61,62].

Furthermore, the interplay between the altered microbiome and hypergastrinemia remains an area of emerging interest [63,64]. Some microbial species have been shown to modulate enteroendocrine cell function and gastrin secretion, potentially amplifying the hypergastrinemic state that drives ECL cell proliferation. Conversely, chronic hypergastrinemia itself may influence the composition of the gastric microbiome by altering mucosal secretions and immune surveillance mechanisms [24,64].

The pro-inflammatory environment fostered by dysbiosis may also contribute to oxidative stress and DNA damage in epithelial cells, creating a genotoxic milieu that facilitates neoplastic transformation [24,65]. This is particularly relevant in the context of ECL cell hyperplasia, where sustained proliferative signaling combined with genotoxic stress could accelerate the progression toward type 1 gNENs.

However, current evidence is limited by methodological issues, including small and heterogeneous cohorts, cross-sectional study designs, variability in sequencing platforms, and the risk of contamination inherent to low-biomass samples. Moreover, it remains unclear whether dysbiosis acts as a driver of disease, a consequence of hypochlorhydria, or merely an epiphenomenon.

Understanding the bidirectional interactions between the gastric microbiome, immune responses, and epithelial dynamics in AIG offers a promising frontier for research. Microbiome-targeted strategies such as probiotics, prebiotics, or fecal microbiota transplantation (FMT) have been proposed, but remain speculative in AIG given the absence of clinical trials.

## 6. Autoimmune Gastritis and Associated Autoimmune Disorder

AIG rarely presents as an isolated condition and is often part of a broader autoimmune spectrum. Epidemiological studies show that up to one-third of patients with AIG develop additional autoimmune diseases, most commonly autoimmune thyroid disease, type 1 diabetes mellitus, vitiligo, and Addison’s disease [4,7]. This clustering is particularly evident within autoimmune polyendocrine syndromes, where AIG coexists with multiple organ-specific disorders.

Shared genetic backgrounds contribute to this overlap, especially HLA-DR3 and HLA-DR4 haplotypes, which predispose to both AIG and other autoimmune conditions [66]. Beyond HLA, polymorphisms in immune-regulatory genes, such as CTLA4 and PTPN22, have been associated with increased susceptibility to concurrent autoimmunity [67]. Immunologically, a breakdown of central tolerance in the thymus and defective peripheral regulatory T-cell function are thought to underpin the coexistence of autoreactivity against different organ-specific antigens.

Clinically, the association with autoimmune thyroid disease (Hashimoto’s thyroiditis or Graves’ disease) is the most frequent and may precede or follow the diagnosis of AIG [68,69]. The link with type 1 diabetes mellitus is well established, and AIG should be considered in diabetic patients presenting with unexplained anemia [70]. Less commonly, associations with celiac disease, autoimmune diseases, and vitiligo have been described, suggesting a common immunogenetic background [4,7,71].

Recognizing the coexistence of AIG with other autoimmune diseases is clinically important. First, the presence of multiple autoimmune conditions may modify the phenotype and course of AIG. Second, systematic screening for thyroid dysfunction, glycemic abnormalities, and other autoimmune manifestations is recommended once AIG is diagnosed. This integrated approach facilitates early recognition, timely treatment, and improved long-term outcomes for affected patients.

## 7. Prognosis and Long-Term Outcomes

The prognosis of AIG is heterogeneous and largely determined by the degree of parietal cell loss and its clinical consequences (Table 1). In many patients, progression is indolent, but the cumulative impact of vitamin B12 deficiency, iron malabsorption, and the risk of neoplastic transformation requires long-term surveillance [1,4,18,72,73].

From a hematologic perspective, pernicious anemia remains the most clinically relevant outcome, with potential complications including irreversible neurological damage if unrecognized or untreated [74]. Iron deficiency may precede cobalamin depletion, particularly in younger patients, and contributes to fatigue and impaired quality of life [75,76].

The strongest prognostic concern relates to the increased risk of gastric neoplasia. Chronic hypergastrinemia promotes ECL cell hyperplasia, which in a subset of patients progresses to type 1 gNENs [15,19,77]. Although gNENs associated with AIG are generally indolent and carry a favorable prognosis [78], their multifocality and tendency for recurrence require endoscopic monitoring [79,80]. In parallel, the risk of gastric adenocarcinoma, although lower than historically estimated, remains elevated compared to the general population [58,77,81,82].

Overall, long-term management should integrate the correction of nutritional deficiencies [76] with structured endoscopic surveillance [72,83]. Current guidelines recommend lifelong follow-up, tailored to individual risk factors such as family history, coexisting autoimmune disorders, and the presence of premalignant lesions [5,72]. Future studies are needed to refine risk stratification and to identify biomarkers capable of predicting disease course and neoplastic transformation.

## 8. Therapeutic Perspectives

The therapeutic landscape of AIG and its sequelae, particularly ECL cell hyperplasia and type 1 gNENs, remains largely empirical and symptom-driven. Current management relies on vitamin B12 supplementation, endoscopic surveillance, and the treatment of established neoplasia by endoscopic or surgical resection. Nevertheless, advances in the understanding of the molecular pathways underpinning parietal cell loss and ECL cell proliferation are paving the way for targeted therapeutic approaches [84] (Table 2).

One of the most promising strategies involves pharmacological modulation of the hypergastrinemia–CCK2R axis. The gastrin/CCK2 receptor antagonist netazepide is the only drug tested in clinical trials for AIG-related type 1 gNENs, where it reduced ECL hyperplasia and induced regression of lesions [84]. By blocking the trophic effect of gastrin, these agents effectively attenuate the proliferative drive on ECL cells and may reduce neoplastic risk [85]. While long-term data are still needed, netazepide represents the only agent so far tested in AIG patients with type 1 gNENs.

Several other strategies remain at a preclinical or conceptual stage, supported largely by mechanistic or animal data. Beyond CCK2R antagonism, pathway-specific inhibitors under development in oncology (e.g., ERK, PI3K, and STAT3 inhibitors) might theoretically be repurposed for AIG-related lesions, although no clinical data are available. In particular, small molecule ERK and PI3K inhibitors, already in clinical development for gastric and other solid tumors, could theoretically be repurposed to restrain ECL cell hyperplasia in AIG [86]. Similarly, STAT3 antagonists may counteract the proliferative and survival signals driving ECL cell hyperplasia [87]. However, given the typically indolent behavior of type 1 gNENs, careful safety evaluation is required before translation to clinical practice. Combination strategies, such as pairing CCK2R antagonists with inhibitors of downstream signaling cascades, remain speculative but could theoretically enhance efficacy and limit resistance.

Targeting intrinsic epithelial stress responses also represents a therapeutic opportunity. Agents targeting epithelial stress pathways, such as modulators of ER stress or enhancers of the UPR, could protect parietal cells from apoptosis, but evidence remains confined to preclinical models [88]. Small molecule chaperones, such as tauroursodeoxycholic acid (TUDCA), have shown promise in reducing ER stress in preclinical models of epithelial injury [89,90] and other chronic inflammatory diseases [91]. These targets could be explored in the context of AIG, but they still remain untested.

Similarly, enhancing autophagic flux in gastric epithelial cells might mitigate cellular vulnerability to inflammation-induced damage. Pharmacological activators of autophagy, including mTOR inhibitors or AMPK activators, may restore cellular homeostasis and preserve parietal cell function, but their systemic effects raise safety concerns [92].

Immunomodulatory approaches, such as restoring Treg function or low-dose IL-2 therapy, could theoretically halt autoimmune aggression, but remain experimental and untested in AIG. Low-dose IL-2 therapy, which preferentially expands Tregs, or the use of checkpoint inhibitors targeting co-stimulatory pathways involved in autoimmunity, may provide innovative strategies, though their application in AIG requires rigorous investigation [93].

Given the emerging role of the gastric microbiome in modulating mucosal immunity and epithelial dynamics, microbiome-targeted interventions have been proposed. Probiotics to restore microbial balance, prebiotics to promote the growth of beneficial bacteria, or even FMT could theoretically reconstitute a protective microbial community. These approaches might synergize with other therapies to reduce inflammation and restore gastric homeostasis, but no clinical trials have yet been conducted in AIG [94].

An integrative therapeutic approach could also arise from simultaneously targeting the immune system, epithelial stress responses, and the altered microbiome. For example, strategies aimed at restoring Treg-mediated tolerance (e.g., low-dose IL-2) may be combined with agents that alleviate ER stress (such as TUDCA) or enhance autophagy, thereby reducing parietal cell vulnerability. In parallel, modulation of the gastric microbiome with probiotics or other microbiota-directed interventions could help dampen inflammation and promote epithelial stability. Leveraging this interplay in a coordinated fashion may offer a more rational and effective strategy than focusing on single mechanisms in isolation.

Despite these promising approaches, several barriers have hindered the translation of preclinical targets into clinical trials. First, the rarity and usually indolent course of type 1 gNENs limit patient recruitment and reduce the perceived need for interventional studies. Second, the absence of validated biomarkers hampers risk stratification and the identification of patients who might benefit from targeted therapies. Third, long-term modulation of immune, autophagy, or stress–response pathways raises safety concerns in a condition that is often asymptomatic or slowly progressive. Finally, limited commercial interest in rare autoimmune gastric disorders reduces the incentive for pharmaceutical investment. These limitations explain why, despite extensive mechanistic insights, netazepide remains the only agent tested in controlled clinical settings.

A further complexity emerges from recent evidence on the dual role of the gastrin–CCK2R axis. A recent study demonstrated that hypergastrinemia can expand CCK2R^+^ isthmus progenitors, accelerate ulcer healing, and even mitigate preneoplastic changes in models of *H. pylori* gastritis and carcinogen exposure [55]. These findings indicate that gastrin signaling is context-dependent, with both pathogenic and regenerative roles, complicating the rationale for long-term CCK2R blockade. This duality may partly explain the slow clinical translation of pathway-targeted therapies: interventions blocking gastrin or CCK2R must balance suppression of ECL hyperplasia with preservation of mucosal repair and host defense.

Translation of mechanistic insights into therapies remains challenging. Until disease-modifying therapies become available, surveillance remains the cornerstone of patient management. Endoscopic monitoring and biomarker assessment are essential for the early detection of neoplastic progression in AIG patients. The identification of molecular biomarkers predictive of ECL cell dysplasia or transformation to gNENs would enhance risk stratification and guide personalized interventions [95].

## 9. Conclusions

**Figure 1:**
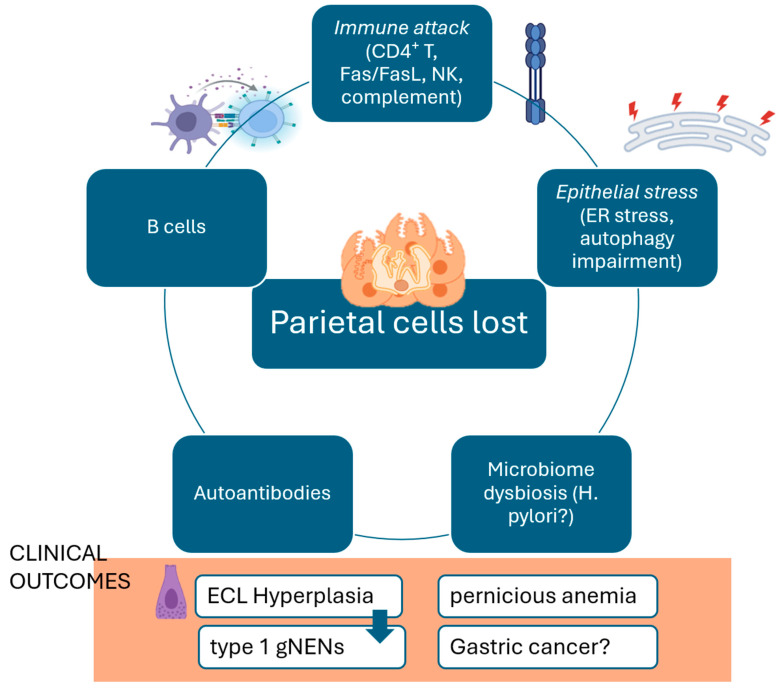
Immune-mediated cytotoxicity (CD4^+^ T-cells, Fas/FasL, NK cells, complement), epithelial stress (ER stress, impaired autophagy), and microbiome dysbiosis (e.g., H. pylori) converge on parietal cell loss. B cells and autoantibodies amplify the autoimmune response. The resulting hypochlorhydria leads to hypergastrinemia, driving enterochromaffin-like (ECL) cell hyperplasia and type 1 gastric neuroendocrine neoplasms (gNENs), while intrinsic factor deficiency causes pernicious anemia.

Parietal cell loss and ECL cell hyperplasia in AIG result from a complex interplay of immune-mediated cytotoxicity, intrinsic epithelial stress responses, and trophic hypergastrinemia-driven signaling. The convergence of autoimmune aggression, intrinsic epithelial stress responses, and chronic hypergastrinemia creates a permissive environment for neoplastic transformation, particularly the development of type 1 gNENs (Figure 1) [15,19,96].

A comprehensive understanding of the molecular and cellular mechanisms underlying parietal cell apoptosis, ECL cell proliferation, and their modulation by the gastric microbiome is critical for advancing clinical management [46]. Dysregulated stress–response and trophic signaling pathways not only explain the molecular cascade of AIG but also explain its main clinical sequelae. Excessive parietal cell apoptosis driven by ER stress and impaired autophagy culminates in hypochlorhydria and intrinsic factor deficiency, the basis of pernicious anemia, whereas persistent activation of ERK/MAPK, PI3K/Akt, and STAT3 sustains ECL cell proliferation and survival, fostering progression toward type 1 gNENs.

These mechanistic insights reveal potential therapeutic targets—such as the ERK/MAPK, PI3K/Akt, and STAT3 cascades [97,98,99,100,101]. Furthermore, recognizing the influence of dysbiosis in perpetuating inflammation and altering epithelial dynamics underscores the need to integrate microbiome-focused strategies into the therapeutic arsenal [24,64]. Among emerging targeted strategies, only CCK2 receptor antagonists such as netazepide have been tested in clinical trials [84], whereas ER stress modulators [89,90], autophagy enhancers [102], and immunomodulatory agents remain at a preclinical or conceptual stage.

Future therapeutic development will likely require integrated strategies that combine immune tolerance restoration, epithelial protection, and microbiome modulation, addressing the multifactorial nature of AIG pathogenesis. In parallel, research should prioritize the identification of predictive biomarkers for neoplastic progression, the design of personalized treatment paradigms, and the execution of well-powered clinical trials.

Finally, significant gaps persist in our understanding of AIG. The contribution of underexplored immune populations (e.g., Tregs, dendritic cells, innate lymphoid cells), the validation of epithelial stress pathways in patient-derived models, and the need for longitudinal microbiome studies remain key unanswered questions. Addressing these gaps will be essential to move beyond descriptive knowledge and toward precision interventions, ultimately improving prognosis and quality of life for patients with AIG.

## Figures and Tables

**Table 1:** Molecular Pathways and Cellular Outcomes in AIG.

Molecular Pathway	Stimuli/Trigger	Primary Cellular Outcome	Relevance to AIG Pathogenesis
ERK/MAPK	Gastrin via CCK2R	ECL proliferation	Promotes hyperplasia and neoplastic risk
PI3K/Akt	Gastrin, growth factors	Enhanced survival, anti-apoptosis	Maintains expanded ECL pool
STAT3	Cytokines, PI3K/Akt crosstalk	Proliferation, angiogenesis	Facilitates gNEN progression
PERK–CHOP (ER stress)	Inflammation, misfolded proteins	Parietal cell apoptosis	Links immune attack to cell loss
Fas/FasL	T-cell/NK engagement	Caspase-8–mediated apoptosis	Direct parietal cytotoxicity
Autophagy	Stress, nutrient deprivation	Organelle clearance	Normally protective; impaired in AIG
TLR signaling	Microbial PAMPs	Innate immune activation	Perpetuates mucosal inflammation

**Table 2:** Therapeutic strategies in AIG in Autoimmune Gastritis.

Strategy	Target/Mechanism	Current Status
Vitamin B12 supplementation	Corrects anemia	Clinical standard
Netazepide (CCK2R antagonist)	Blocks gastrin-driven ECL hyperplasia	Clinical trials completed
ER stress modulators (e.g., TUDCA)	Reduce parietal cell apoptosis	Preclinical
Autophagy enhancers	Promote epithelial resilience	Preclinical
Immunomodulators (e.g., Treg therapies)	Restore tolerance	Theoretical
Microbiome modulation	Correct dysbiosis	Preclinical/theoretical

## Data Availability

No new data were created or analyzed in this study. Data sharing does not apply to this article.

## References

[1] Lenti M.V., Rugge M., Lahner E., Miceli E., Toh B.H., Genta R.M., De Block C., Hershko C., Di Sabatino A. (2020). Autoimmune gastritis. Nat. Rev. Dis. Primers.

[2] Soykan İ., Er R.E., Baykara Y., Kalkan C. (2024). Unraveling the Mysteries of Autoimmune Gastritis. Turk. J. Gastroenterol..

[3] Castellana C., Eusebi L.H., Dajti E., Iascone V., Vestito A., Fusaroli P., Fuccio L., D’errico A., Zagari R.M. (2024). Autoimmune Atrophic Gastritis: A Clinical Review. Cancers.

[4] Massironi S., Zilli A., Elvevi A., Invernizzi P. (2019). The changing face of chronic autoimmune atrophic gastritis: An updated comprehensive perspective. Autoimmun. Rev..

[5] Shah S.C., Piazuelo M.B., Kuipers E.J., Li D. (2021). AGA Clinical Practice Update on the Diagnosis and Management of Atrophic Gastritis: Expert Review. Gastroenterology.

[6] Vavallo M., Cingolani S., Cozza G., Schiavone F.P., Dottori L., Palumbo C., Lahner E. (2024). Autoimmune Gastritis and Hypochlorhydria: Known Concepts from a New Perspective. Int. J. Mol. Sci..

[7] Massironi S. (2025). Autoimmune gastritis: An organ-specific disease or a model of systemic autoimmunity? Parallels, divergences, and emerging insights. Expert Rev. Gastroenterol. Hepatol..

[8] Taylor L., McCaddon A., Wolffenbuttel B.H.R. (2024). Creating a Framework for Treating Autoimmune Gastritis-The Case for Replacing Lost Acid. Nutrients.

[9] Toh B.H., Sentry J.W., Alderuccio F. (2000). The causative H+/K+ ATPase antigen in the pathogenesis of autoimmune gastritis. Immunol. Today.

[10] Iwamuro M., Tanaka T., Otsuka M. (2023). Update in Molecular Aspects and Diagnosis of Autoimmune Gastritis. Curr. Issues Mol. Biol..

[11] De Prado Á., Cal-Sabater P., Fiz-López A., Izquierdo S., Corrales D., Pérez-Cózar F., H-Vázquez J., Arribas-Rodríguez E., Perez-Segurado C., Muñoz Á.M. (2024). Complex immune network and regional consistency in the human gastric mucosa revealed by high-resolution spectral cytometry. Sci. Rep..

[12] Jiao Y., Yan Z., Yang A. (2023). The Roles of Innate Lymphoid Cells in the Gastric Mucosal Immunology and Oncogenesis of Gastric Cancer. Int. J. Mol. Sci..

[13] Nouari W., Aribi M. (2025). Innate lymphoid cells, immune functional dynamics, epithelial parallels, and therapeutic frontiers in infections. Int. Rev. Immunol..

[14] Waldum H., Mjønes P. (2020). Towards Understanding of Gastric Cancer Based upon Physiological Role of Gastrin and ECL Cells. Cancers.

[15] Massironi S., Gallo C., Elvevi A., Stegagnini M., Coltro L.A., Invernizzi P. (2023). Incidence and prevalence of gastric neuroendocrine tumors in patients with chronic atrophic autoimmune gastritis. World J. Gastrointest. Oncol..

[16] Yu Z., Wang A., Hu C., Yu T., Chen J. (2022). Type-1 Grade 2 Multi-Focal Gastric Neuroendocrine Tumors Secondary to Chronic Autoimmune Gastritis. Front. Med..

[17] Brown P., Tetali B., Suresh S., Varma A. (2021). Progression From Antral G-Cell Hyperplasia to Gastric Neuroendocrine Tumor in a Patient with Autoimmune Gastritis. ACG Case Rep. J..

[18] Miceli E., Lenti M.V., Gentile A., Gambini G., Petrucci C., Pitotti L., Mengoli C., Di Stefano M., Vanoli A., Luinetti O. (2023). Long-term natural history of autoimmune gastritis: Results from a prospective, monocentric series. Am. J. Gastroenterol..

[19] Massironi S., Sciola V., Spampatti M.P., Peracchi M., Conte D. (2009). Gastric carcinoids: Between underestimation and overtreatment. World J. Gastroenterol..

[20] Peracchi M., Gebbia C., Basilisco G., Quatrini M., Tarantino C., Vescarelli C., Massironi S., Conte D. (2005). Plasma chromogranin A in patients with autoimmune chronic atrophic gastritis, enterochromaffin-like cell lesions and gastric carcinoids. Eur. J. Endocrinol..

[21] Sheng W., Malagola E., Nienhüser H., Zhang Z., Kim W., Zamechek L., Sepulveda A., Hata M., Hayakawa Y., Zhao C.-M. (2020). Hypergastrinemia Expands Gastric ECL Cells Through CCK2R^+^ Progenitor Cells via ERK Activation. Cell Mol. Gastroenterol. Hepatol..

[22] Zhang T., Tang X. (2025). Beyond metaplasia: Unraveling the complex pathogenesis of autoimmune atrophic gastritis and its implications for gastric cancer risk. Qjm.

[23] Goldenring J.R., Nam K.T. (2010). Oxyntic atrophy, metaplasia, and gastric cancer. Prog. Mol. Biol. Transl. Sci..

[24] Conti L., Annibale B., Lahner E. (2020). Autoimmune Gastritis and Gastric Microbiota. Microorganisms.

[25] D’ELios M.M., Amedei A., Azzurri A., Benagiano M., Del Prete G., Bergman M.P., Vandenbroucke-Grauls C.M., Appelmelk B.J. (2005). Molecular specificity and functional properties of autoreactive T-cell response in human gastric autoimmunity. Int. Rev. Immunol..

[26] Toh B.H., van Driel I.R., Gleeson P.A. (1992). Autoimmune gastritis: Tolerance and autoimmunity to the gastric H+/K+ ATPase (proton pump). Autoimmunity.

[27] Suleymanov Z. (2003). Expression of class I and II MHC receptors in Helicobacter pylori-positive patients with active gastritis and duodenal ulcer. Turk. J. Gastroenterol..

[28] Toh B.H., A Gleeson P., Simpson R.J., Moritz R.L., Callaghan J.M., Goldkorn I., Jones C.M., Martinelli T.M., Mu F.T., Humphris D.C. (1990). The 60- to 90-kDa parietal cell autoantigen associated with autoimmune gastritis is a beta subunit of the gastric H+/K(+)-ATPase (proton pump). Proc. Natl. Acad. Sci. USA.

[29] Lahner E., Brigatti C., Marzinotto I., Carabotti M., Scalese G., Davidson H.W., Wenzlau J.M., Bosi E., Piemonti L., Annibale B. (2017). Luminescent Immunoprecipitation System (LIPS) for Detection of Autoantibodies Against ATP4A and ATP4B Subunits of Gastric Proton Pump H+,K+-ATPase in Atrophic Body Gastritis Patients. Clin. Transl. Gastroenterol..

[30] Alderuccio F., Murphy K., Biondo M., Field J., Toh B.H. (2005). Reversing the autoimmune condition: Experience with experimental autoimmune gastritis. Int. Rev. Immunol..

[31] Alderuccio F., Sentry J.W., Marshall A.C., Biondo M., Toh B.H. (2002). Animal models of human disease: Experimental autoimmune gastritis--a model for autoimmune gastritis and pernicious anemia. Clin. Immunol..

[32] Zhang Z., Zhu T., Zhang L., Xing Y., Yan Z., Li Q. (2023). Critical influence of cytokines and immune cells in autoimmune gastritis. Autoimmunity.

[33] Mommersteeg M., Simovic I., Yu B., van Nieuwenburg S., I M.B., Doukas M., Kuipers E., Spaander M., Peppelenbosch M., Castaño-Rodríguez N. (2022). Autophagy mediates ER stress and inflammation in Helicobacter pylori-related gastric cancer. Gut Microbes.

[34] Gundu C., Arruri V.K., Sherkhane B., Khatri D.K., Singh S.B. (2022). GSK2606414 attenuates PERK/p-eIF2α/ATF4/CHOP axis and augments mitochondrial function to mitigate high glucose induced neurotoxicity in N2A cells. Curr. Res. Pharmacol. Drug Discov..

[35] Huang J., Wan L., Lu H., Li X. (2018). High expression of active ATF6 aggravates endoplasmic reticulum stress-induced vascular endothelial cell apoptosis through the mitochondrial apoptotic pathway. Mol. Med. Rep..

[36] Rudi J., Kuck D., Strand S., von Herbay A., Mariani S.M., Krammer P.H., Galle P.R., Stremmel W. (1998). Involvement of the CD95 (APO-1/Fas) receptor and ligand system in Helicobacter pylori-induced gastric epithelial apoptosis. J. Clin. Investig..

[37] Watanabe R., Fujii H., Shirai T., Saito S., Ishii T., Harigae H. (2014). Autophagy plays a protective role as an anti-oxidant system in human T cells and represents a novel strategy for induction of T-cell apoptosis. Eur. J. Immunol..

[38] He Q., Liu M., Rong Z., Liang H., Xu X., Sun S., Lei Y., Li P., Meng H., Zheng R. (2022). Rebamipide attenuates alcohol-induced gastric epithelial cell injury by inhibiting endoplasmic reticulum stress and activating autophagy-related proteins. Eur. J. Pharmacol..

[39] Chen Y.-C., Lin I.-C., Su M.-C., Hsu P.-Y., Hsiao C.-C., Hsu T.-Y., Liou C.-W., Chen Y.-M., Chin C.-H., Wang T.-Y. (2023). Autophagy impairment in patients with obstructive sleep apnea modulates intermittent hypoxia-induced oxidative stress and cell apoptosis via hypermethylation of the ATG5 gene promoter region. Eur. J. Med. Res..

[40] Chen X., Liu R., Liu X., Xu C., Wang X. (2018). L-ascorbic Acid-2-Glucoside inhibits Helicobacter pylori-induced apoptosis through mitochondrial pathway in Gastric Epithelial cells. Biomed. Pharmacother..

[41] Calvino-Fernández M., Benito-Martínez S., Parra-Cid T. (2008). Oxidative stress by Helicobacter pylori causes apoptosis through mitochondrial pathway in gastric epithelial cells. Apoptosis.

[42] Zhao S., Wang H., Nie Y., Mi Q., Chen X., Hou Y. (2012). Midkine upregulates MICA/B expression in human gastric cancer cells and decreases natural killer cell cytotoxicity. Cancer Immunol. Immunother..

[43] Rodella L., Rezzani R., Zauli G., Mariani A.R., Rizzoli R., Vitale M. (1998). Apoptosis induced by NK cells is modulated by the NK-active cytokines IL-2 and IL-12. Int. Immunol..

[44] Poggi A., Benelli R., Venè R., Costa D., Ferrari N., Tosetti F., Zocchi M.R. (2019). Human Gut-Associated Natural Killer Cells in Health and Disease. Front. Immunol..

[45] Della Bella C., Antico A., Panozzo M.P., Capitani N., Petrone L., Benagiano M., D’eLios S., Sparano C., Azzurri A., Pratesi S. (2022). Gastric Th17 Cells Specific for H(+)/K(+)-ATPase and Serum IL-17 Signature in Gastric Autoimmunity. Front. Immunol..

[46] Judd L.M., Gleeson P.A., Toh B.H., van Driel I.R. (1999). Autoimmune gastritis results in disruption of gastric epithelial cell development. Am. J. Physiol..

[47] Torbenson M., Abraham S.C., Boitnott J., Yardley J.H., Wu T.T. (2002). Autoimmune gastritis: Distinct histological and immunohistochemical findings before complete loss of oxyntic glands. Mod. Pathol..

[48] Watanabe H., Yoneda S., Motoyama Y., Mukai K., Okuno Y., Kozawa J., Nishizawa H., Maeda N., Otsuki M., Matsuoka T.-A. (2020). Marked Hypergastrinemia with G-cell Hyperplasia in Two Autoimmune Gastritis Patients. Intern. Med..

[49] Waldum H.L., Brenna E., Sandvik A.K. (1998). Relationship of ECL cells and gastric neoplasia. Yale J. Biol. Med..

[50] Prinz C., Scott D.R., Hurwitz D., Helander H.F., Sachs G. (1994). Gastrin effects on isolated rat enterochromaffin-like cells in primary culture. Am. J. Physiol..

[51] Chen D., Zhao C.M., Al-Haider W., Håkanson R., Rehfeld J.F., Kopin A.S. (2002). Differentiation of gastric ECL cells is altered in CCK(2) receptor-deficient mice. Gastroenterology.

[52] Shamburek R.D., Schubert M.L. (1992). Control of gastric acid secretion. Histamine H2-receptor antagonists and H+K(+)-ATPase inhibitors. Gastroenterol. Clin. N. Am..

[53] Obrink K.J. (1991). Histamine and gastric acid secretion. A review. Scand. J. Gastroenterol. Suppl..

[54] Nguyen T.L., Khurana S.S., Bellone C.J., Capoccia B.J., Sagartz J.E., Kesman R.A., Jr et a.l. (2013). Autoimmune gastritis mediated by CD4+ T cells promotes the development of gastric cancer. Cancer Res..

[55] Zheng B., Kobayashi H., Tu R., Huang K., Zhi X., Lian G., Wu F., Qian J., Ochiai Y., Waterbury Q.T. (2025). Gastrin-dependent expansion of Cck2r(+) corpus progenitors accelerates ulcer healing and inhibits gastric dysplasia. Gut.

[56] Zhang A., Niu L., Ni Y., Liu W., Gao X., Chang L., Cao P. (2025). STAT3 inhibition mitigates experimental autoimmune gastritis by restoring Th17/Treg immune balance. Immunol. Res..

[57] Takeuchi C., Sato J., Yamashita S., Sasaki A., Akahane T., Aoki R., Yamamichi M., Liu Y.-Y., Ito M., Furuta T. (2022). Autoimmune gastritis induces aberrant DNA methylation reflecting its carcinogenic potential. J. Gastroenterol..

[58] Arai J., Niikura R., Hayakawa Y., Suzuki N., Hirata Y., Ushiku T., Fujishiro M. (2022). Clinicopathological Features of Gastric Cancer with Autoimmune Gastritis. Biomedicines.

[59] Takahashi Y., Uno K., Iijima K., Abe Y., Koike T., Asano N., Asanuma K., Shimosegawa T. (2018). Acidic bile salts induces mucosal barrier dysfunction through let-7a reduction during gastric carcinogenesis after Helicobacter pylori eradication. Oncotarget.

[60] McColl K.E. (2012). The elegance of the gastric mucosal barrier: Designed by nature for nature. Gut.

[61] Chen B., Sun L., Zhang X. (2017). Integration of microbiome and epigenome to decipher the pathogenesis of autoimmune diseases. J. Autoimmun..

[62] Rogier R., Koenders M.I., Abdollahi-Roodsaz S. (2015). Toll-like receptor mediated modulation of T cell response by commensal intestinal microbiota as a trigger for autoimmune arthritis. J. Immunol. Res..

[63] Huo S., Lv K., Han L., Zhao Y., Jiang J. (2025). Gut microbiota in gastric cancer: From pathogenesis to precision medicine. Front. Microbiol..

[64] Rajilic-Stojanovic M., Figueiredo C., Smet A., Hansen R., Kupcinskas J., Rokkas T., Andersen L., Machado J.C., Ianiro G., Gasbarrini A. (2020). Systematic review: Gastric microbiota in health and disease. Aliment. Pharmacol. Ther..

[65] Ferreira R.M., Pereira-Marques J., Pinto-Ribeiro I., Costa J.L., Carneiro F., Machado J.C., Figueiredo C. (2018). Gastric microbial community profiling reveals a dysbiotic cancer-associated microbiota. Gut.

[66] Oksanen A.M., Haimila K.E., Rautelin H.I., Partanen J.A. (2010). Immunogenetic characteristics of patients with autoimmune gastritis. World J. Gastroenterol..

[67] Brorsson C.A., Pociot F. (2015). Shared Genetic Basis for Type 1 Diabetes, Islet Autoantibodies, and Autoantibodies Associated with Other Immune-Mediated Diseases in Families with Type 1 Diabetes. Diabetes Care.

[68] Cellini M., Santaguida M.G., Virili C., Capriello S., Brusca N., Gargano L., Centanni M. (2017). Hashimoto’s Thyroiditis and Autoimmune Gastritis. Front. Endocrinol..

[69] Boutzios G., Koukoulioti E., Goules A.V., Kalliakmanis I., Giovannopoulos I., Vlachoyiannopoulos P., Moutsopoulos H.M., Tzioufas A.G. (2022). Hashimoto Thyroiditis, Anti-Parietal Cell Antibodies: Associations with Autoimmune Diseases and Malignancies. Front. Endocrinol..

[70] De Block C.E., De Leeuw I.H., Van Gaal L.F. (2008). Autoimmune gastritis in type 1 diabetes: A clinically oriented review. J. Clin. Endocrinol. Metab..

[71] Massironi S., Dispinzieri G., Rossi A., Cristoferi L., Lenti M.V., Gerussi A., Elvevi A., Carbone M., Bonfichi A., Di Sabatino A. (2025). Immunological and clinical overlap between autoimmune gastritis and autoimmune liver diseases: A prospective cohort study. Front. Immunol..

[72] Lahner E., Zagari R.M., Zullo A., Di Sabatino A., Meggio A., Cesaro P., Lenti M.V., Annibale B., Corazza G.R. (2019). Chronic atrophic gastritis: Natural history, diagnosis and therapeutic management. A position paper by the Italian Society of Hospital Gastroenterologists and Digestive Endoscopists [AIGO], the Italian Society of Digestive Endoscopy [SIED], the Italian Society of Gastroenterology [SIGE], and the Italian Society of Internal Medicine [SIMI]. Dig. Liver Dis..

[73] Lenti M.V., Broglio G., Di Sabatino A. (2023). Unravelling the risk of developing gastric cancer in autoimmune gastritis. Gut.

[74] Toh B.H., Alderuccio F. (2004). Pernicious anaemia. Autoimmunity.

[75] Rogez J., Urbanski G., Vinatier E., Lavigne C., Emmanuel L., Dupin I., Ravaiau C., Lacombe V. (2024). Iron deficiency in pernicious anemia: Specific features of iron deficient patients and preliminary data on response to iron supplementation. Clin. Nutr..

[76] Cavalcoli F., Zilli A., Conte D., Massironi S. (2017). Micronutrient deficiencies in patients with chronic atrophic autoimmune gastritis: A review. World J. Gastroenterol..

[77] Chen C., Yang Y., Li P., Hu H. (2023). Incidence of Gastric Neoplasms Arising from Autoimmune Metaplastic Atrophic Gastritis: A Systematic Review and Case Reports. J. Clin. Med..

[78] Dell’UNto E., Mandair D., Riding G., Rimondi A., Rinzivillo M., Esposito G., Luong T.V., Lahner E., Watkins J., Annibale B. (2025). The indolent nature of type 1 gastric neuroendocrine tumors under 1 cm. Dig. Liver Dis..

[79] Massironi S., Zilli A., Fanetti I., Ciafardini C., Conte D., Peracchi M. (2015). Intermittent treatment of recurrent type-1 gastric carcinoids with somatostatin analogues in patients with chronic autoimmune atrophic gastritis. Dig. Liver Dis..

[80] Merola E., Sbrozzi-Vanni A., Panzuto F., D’aMbra G., Di Giulio E., Pilozzi E., Capurso G., Lahner E., Bordi C., Annibale B. (2012). Type I gastric carcinoids: A prospective study on endoscopic management and recurrence rate. Neuroendocrinology.

[81] Lahner E., Annibale B., Dilaghi E., Millado C.L., Lenti M.V., Di Sabatino A., Miceli E., Massironi S., Zucchini N., Cannizzaro R. (2025). Clinical and Endoscopic-Histological Features of Multifocal and Corpus-Restricted Atrophic Gastritis Patients with Non-Cardia Gastric Cancer or Dysplasia: A Multicenter, Cross-Sectional Study. Clin. Transl. Gastroenterol..

[82] Rugge M., Bricca L., Guzzinati S., Sacchi D., Pizzi M., Savarino E., Farinati F., Zorzi M., Fassan M., Tos A.P.D. (2023). Autoimmune gastritis: Long-term natural history in naïve Helicobacter pylori-negative patients. Gut.

[83] Esposito G., Dilaghi E., Cazzato M., Pilozzi E., Conti L., Carabotti M., Di Giulio E., Annibale B., Lahner E. (2021). Endoscopic surveillance at 3 years after diagnosis, according to European guidelines, seems safe in patients with atrophic gastritis in a low-risk region. Dig. Liver Dis..

[84] Boyce M., van den Berg F., Mitchell T., Darwin K., Warrington S. (2017). Randomised trial of the effect of a gastrin/CCK_2_ receptor antagonist on esomeprazole-induced hypergastrinaemia: Evidence against rebound hyperacidity. Eur. J. Clin. Pharmacol..

[85] Kidd M., Siddique Z.-L., Drozdov I., Gustafsson B., Camp R., Black J., Boyce M., Modlin I. (2010). The CCK(2) receptor antagonist, YF476, inhibits Mastomys ECL cell hyperplasia and gastric carcinoid tumor development. Regul. Pept..

[86] Yuan W., Shi Y., Dai S., Deng M., Zhu K., Xu Y., Chen Z., Xu Z., Zhang T., Liang S. (2024). The role of MAPK pathway in gastric cancer: Unveiling molecular crosstalk and therapeutic prospects. J. Transl. Med..

[87] Gharibi T., Babaloo Z., Hosseini A., Abdollahpour-Alitappeh M., Hashemi V., Marofi F., Nejati K., Baradaran B. (2020). Targeting STAT3 in cancer and autoimmune diseases. Eur. J. Pharmacol..

[88] Deka D., D’Incà R., Sturniolo G.C., Das A., Pathak S., Banerjee A. (2022). Role of ER Stress Mediated Unfolded Protein Responses and ER Stress Inhibitors in the Pathogenesis of Inflammatory Bowel Disease. Dig. Dis. Sci..

[89] Zhao J., Hao S., Chen Y., Ye X., Fang P., Hu H. (2024). Tauroursodeoxycholic acid liposome alleviates DSS-induced ulcerative colitis through restoring intestinal barrier and gut microbiota. Colloids Surf. B Biointerfaces.

[90] Pan D., Wang J., Ye H., Qin Y., Xu S., Ye G., Shen H. (2024). Tauroursodeoxycholic acid suppresses biliary epithelial cell apoptosis and endoplasmic reticulum stress by miR-107/NCK1 axis in a FXR-dependent manner. Drug Chem. Toxicol..

[91] Lamm V., Deng R., Huang K., Soleymanjahi S., Liu T.-C., Xie Y., Gremida A.K., Deepak P., Chen C.-H., Davidson N.O. (2025). Tauroursodeoxycholic Acid (TUDCA) Reduces ER Stress and Lessens Disease Activity in Ulcerative Colitis. medRxiv.

[92] Lee H., Lim J.W., Kim H. (2020). Effect of Astaxanthin on Activation of Autophagy and Inhibition of Apoptosis in Helicobacter pylori-Infected Gastric Epithelial Cell Line AGS. Nutrients.

[93] McQuaid S.L., Loughran S.T., Power P.A., Maguire P., Szczygiel A., Johnson P.A. (2020). Low-dose IL-2 induces CD56(bright) NK regulation of T cells via NKp44 and NKp46. Clin. Exp. Immunol..

[94] Amoroso C., Perillo F., Strati F., Fantini M.C., Caprioli F., Facciotti F. (2020). The Role of Gut Microbiota Biomodulators on Mucosal Immunity and Intestinal Inflammation. Cells.

[95] Dottori L., Pivetta G., Annibale B., Lahner E. (2023). Update on Serum Biomarkers in Autoimmune Atrophic Gastritis. Clin. Chem..

[96] Nehme F., Rowe K., Palko W., Tofteland N., Salyers W. (2020). Autoimmune metaplastic atrophic gastritis and association with neuroendocrine tumors of the stomach. Clin. J. Gastroenterol..

[97] Lv Y., Chen C., Han M., Tian C., Song F., Feng S., Xu M., Zhao Z., Zhou H., Su W. (2025). CXCL2: A key player in the tumor microenvironment and inflammatory diseases. Cancer Cell Int..

[98] Lv B., Song C., Wu L., Zhang Q., Hou D., Chen P., Yu S., Wang Z., Chu Y., Zhang J. (2015). Netrin-4 as a biomarker promotes cell proliferation and invasion in gastric cancer. Oncotarget.

[99] Pandian J., Ganesan K. (2022). Delineation of gastric tumors with activated ERK/MAPK signaling cascades for the development of targeted therapeutics. Exp. Cell Res..

[100] Xu W., Chen G.-S., Shao Y., Li X.-L., Xu H.-C., Zhang H., Zhu G.-Q., Zhou Y.-C., He X.-P., Sun W.-H. (2013). Gastrin acting on the cholecystokinin2 receptor induces cyclooxygenase-2 expression through JAK2/STAT3/PI3K/Akt pathway in human gastric cancer cells. Cancer Lett..

[101] Morgos D.-T., Stefani C., Miricescu D., Greabu M., Stanciu S., Nica S., Stanescu-Spinu I.-I., Balan D.G., Balcangiu-Stroescu A.-E., Coculescu E.-C. (2024). Targeting PI3K/AKT/mTOR and MAPK Signaling Pathways in Gastric Cancer. Int. J. Mol. Sci..

[102] Chipurupalli S., Samavedam U., Robinson N. (2021). Crosstalk Between ER Stress, Autophagy and Inflammation. Front. Med..

